# Supplementary material for: Nutritional Characterization of Strychnos madagascariensis Fruit Flour Produced by Mozambican Communities and Evaluation of Its Contribution to Nutrient Adequacy
Source: Foods. 2022 Feb 21;11(4):616. doi: 10.3390/foods11040616 (PMC8870968; doi:10.3390/foods11040616)
Supplement: Supplementary file 1 [file foods-11-00616-s001.zip › foods-1602577-supplementary.pdf]

Table S1. Limits of detection (LOD) for the elements analyzed by ICP-MS.

Element	LOD (µg/g)
Al	0.928
As	0.127
Ba	0.091
Be	0.011
Bi	0.009
Cd	0.024
Co	0.020
Cr	0.033
Cs	0.012
Cu	0.233
Li	0.013
Mn	0.041
Ni	0.335
Pb	0.018
Rb	0.021
Se	0.181
Sr	0.036
Tl	0.006
V	0.041
Zn	0.529
